# Tunable superapolar *Lotus-to-Rose* hierarchical nanosurfaces *via* vertical carbon nanotubes driven electrohydrodynamic lithography†
†Electronic supplementary information (ESI) available. See DOI: 10.1039/c6nr08706j



**DOI:** 10.1039/c6nr08706j

**Published:** 2017-01-04

**Authors:** Chiara Busà, Jonathan James Stanley Rickard, Eugene Chun, Yaw Chong, Viroshan Navaratnam, Pola Goldberg Oppenheimer

**Affiliations:** a School of Chemical Engineering , University of Birmingham , Birmingham B15 2TT , UK . Email: GoldberP@bham.ac.uk; b Department of Physics , Cavendish Laboratory , University of Cambridge , Cambridge CB3 0HE , UK

## Abstract

The development of a robust, cost-effective, scalable and simple technique that enables the design and construction of well-controlled large area superhydrophobic surface structures which can be easily tuned from *lotus-leaf* to *rose-petal* state is essential to enable progress in realising the full applied potential of such surfaces. In this study, we introduce the tuneable carbon nanotubes-based electrohydrodynamic lithography (CNT-EHL) to fabricate unique multiscale structured cones and nanohair-like architectures with various periodicities and dimensions, successfully enabling surface energy minimization. The possibility of contact-less lithography *via* the CNT-EHL morphology replication combined with the electric field coupling to smaller self-assembled patterns within the film, provides a way for hierarchical structure control spanning many length scales along with tuneable wetting capabilities. By controlling the hierarchy of micro- to nano cones and spikes, these morphologies provide a range of architectures with sufficient roughness for very low wettability, with the highest contact angle achieved of 173° and their properties can be easily switched between *lotus-leaf* to *rose-petal* behaviour.

## Abstract 2

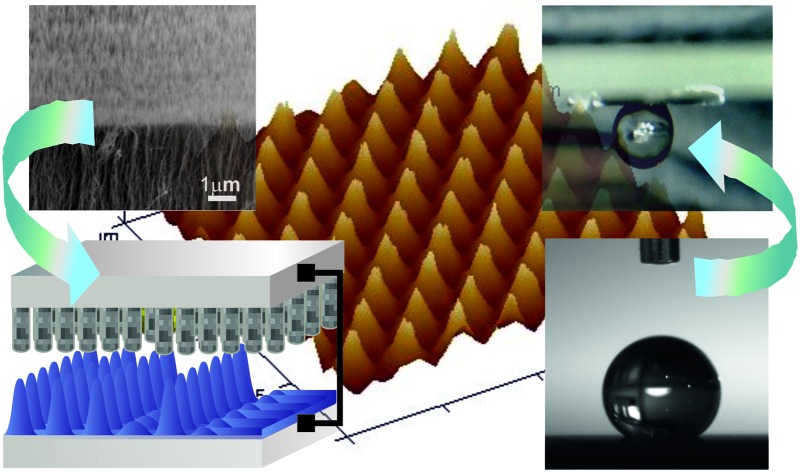
The development of an advanced technique that enables fabrication of superhydrophobic structured surfaces, easily tuneable from *lotus-leaf* to *rose-petal* state is essential to realise the full applied potential of such architectures.

Super-apolar surfaces which exhibit unique self-cleaning properties are very promising for a broad range of applications ranging from coatings for windows, cloths and car windshields, to the anti-corrosive covering for aircraft^1,2^ and marine vessels to antifogging and anti-icing finishes.^3^ Due to the exciting breadth of potential, considerable efforts have been focussed into developing artificially engineered surfaces to biomimic the extreme water repelling properties, notably known as the ‘*Lotus-Leaf*’ effect. The classical Wenzel and Casie–Baxter models describe the correlation between the surface roughness and the wettability properties.^4,5^ While the Wenzel regime explains the intrinsic wetting tendency of a liquid to adhere to the surface after contact, the Casie–Baxter mode refers to the state at which it is energetically more favourable for the water drop to bridge across the top of a composite dual-layered surface structure with air trapped within the asperities. The transitional regime between the Casie–Baxter and Wenzel state usually exhibits high contact angle hysteresis and depends on the dimensions and gaps between the surface roughness structures as well as the chemical hydrophobicity. However more recently, it has been acknowledged that it is important to consider wetting modes beyond the classical and the broadly described Wenzel/Casie–Baxter states recognising existence of not only the ‘*Lotus-Leaf*’ effect but also of a strong adhesion combined with super-hydrophobicity known as ‘*Rose-Petal*’ state^6,7^ and thus, distinguishing wetting regimes of a surface with a single level of roughness and the hierarchical ones (Fig. 1b).

**Fig. 1:**
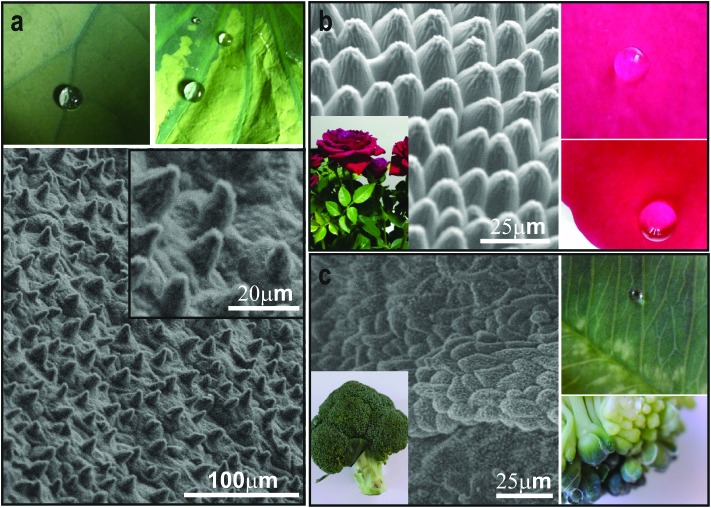
Superhydrophobicity in nature. (a) Optical images of the spherical water drops rolling-off the natural *Nelumbo nucifera* (lotus leaf) and the corresponding scanning electron microscopy (SEM) image of the surface topography of the lotus-leaf with the higher magnification of the lotus surface shown in the inset. (b) SEM image of the structure found on the *rose* petal and the corresponding photograph images of spherical water droplets sticking to the *rose-petal* surface. SEM image of the microstructure found in *broccoli* and the corresponding optical images of its superhydrophobic properties.

The existence of such a spectrum of the wetting states can be understood through the competition of forces acting on the solid–liquid in terms of surface energy, which is inversely proportional to the contact angle adhesion of water molecules to the rough surface as well as impregnation of the hierarchical structures by water or air. For instance, small liquid–solid adhesion typically results in high contact angle and low contact angle hysteresis, facilitating super repellent surfaces. On the other hand, when strong adhesion coexists between molecules of liquid at the same time as low surface energy, surfaces tend to be both superhydrophobic and at the same time sticky. The adhesion hysteresis yields an asymmetry between the wetting and dewetting processes, with higher energy required for the latter. Natural super-hydrophobic systems such as the *Lotus* leaf consists of micron sized papillae spaced apart from each other by a micron scale distance and nano hair like mats on each micro-papillae, yielding the highly apolar surface with very low contact angle hysteresis (Fig. 1a). The hierarchical texture of the natural systems allows enhanced water repellence (Fig. 1) for both static and dynamic conditions. Biological adhesive systems such as for instance Gecko's pads excel in terms of adhesive strength on virtually any surface due to the millions of densely assembled high aspect ratio adhesive setae, which are arranged in a grid-like pattern on the ventral surface of each scansor, branching out into hundreds of nanometer-sized spatular tips, allowing them to deform and adhere to nearly any surface. The red rose petals maintain spherical droplets on their surface which do not roll off even if the petal is turned upside down (Fig. 1b) thus, exhibiting both superhydrophobicity along with high adhesive force with the water. While technologically valuable, both high-density hierarchical fibrillar adhesives and precisely orchestred periodic micro-to-nano super apolar structures are difficult to manufacture in a straightforward manner from a material of choice and no scalable low-cost approach yet exist to create the required tuneable geometries.

The vast majority of previous routes to generate synthetic superhydrophobic morphologies using low surface energy materials with high or low adhesion are based on conventional patterning methods.^8–10^ These are not cost-effective, and are time consuming, often cumbersome and necessitate an accurate combination of multistep processes, thus limiting the scalability of the resulting architectures. For instance, while photolithography^8,11^ is an established method for generating structures optically, it is not a straightforward route to create micro and nanoscale roughness simultaneously due to the sequential photoresist development and film etching processes involved, rendering it essentially a two-dimensional technique, where many steps must be iterated to create a three dimensional structure, it is also limited by the diffraction limit. Alternative approaches such as electron-beam lithography^12^ require many sequential steps, are costly and highly susceptible to beam-drift occurring due to the long exposure times. Replication fidelity using the majority of the techniques employed to fabricate the sought after hierarchical superhydrophobic architectures on substrates with desired chemical and physical properties, is predominantly low and often results in a stochastic mixture of non-periodic and non-optimal packing of the final submicron structures, unlike the ones found in nature, (Fig. 1a–c) and frequently accomplished *via* using hazardous organic compounds and corrosive gases.^13,14^ This in turn, results in inconsistent and partial wetting states on meso and macro scales. Fabrication of multitier morphologies with tuneable wetting exploiting biologically inspired routes, self-assembly processes, templating block-copolymer based approaches and various imprinting techniques are typically limited by poor mechanical stability, mold-pattern distortions, cost, difficulties to manufacture bicontinuous morphologies in all three spatial dimensions and the principal uncontrollability and irreproducibility.^15–21^

Here we demonstrate an unconventional route to reproducibly manufacture scalable nano to micro nanohair-like surfaces as well as hierarchical cone-structure arrays with various curvatures, patterned from vertically aligned carbon nanotube-based master electrodes *via* advanced electrohydrodynamic lithography (CNT-EHL). While electrohydrodynamic patterning was previously used to pattern dielectric, conductive and crystalline materials,^22–25^ this is the first time that vertically aligned CNTs are exploited to generate the master electrodes which are further employed as top masks to generate and control the fabricated morphologies *via* the EHL. This innovative approach allows fabricating a range of reusable CNT-based electrodes with various morphologies and dimensions, enabling direct and tuneable patterning from the material of choice and with no need of functionalisation (*i.e.*, inexpensive, no processing equipment) of robust and highly-reproducible structures which exhibit hexagonal packing symmetry. Importantly, the CNTs generated master electrodes, once coated with a metallic nanloayer, are of a high structural integrity and durability and can be used to consistently produce structures of interest from low-cost materials and therefore, enabling high-reproducibility, cost-efficiency and scalability of the generated nanosurfaces. Moreover, the versatile CNTs-based masks can be easily tailored and tuned in structural dimensions and nano-gaps by simply varying their growth densities. Nanohair-like structures, cones and sharp spiky micro and nano arrays, which are typically difficult to manufacture, are successfully fabricated *via* the CNT-EHL in a single step which can be easily tuned in dimensions, pitch, aspect ratio and the cone tip curvature. Utilising low-energy materials, the CNT-EHL fabricated micro to-nanoscale roughness allows precisely tailoring and controlling hierarchical geometries by adjusting the patterning parameters and thus, significantly influencing the surface wetting properties and mimicking the various regimes found in nature. This method enables tuning and alternating between the *lotus-leaf* and *rose-petal* behaviour due to the controllable experimental approach and the ultimate morphologies generated while patterned from the same initial material. The generated superhydrophobic surfaces with self-cleaning or adhesive properties are promising prospects for both the fundamental research of submicron scale superhydrophobicity and broader applications including, in anti-fouling and microfluidics. CNT-EHL therefore, opens a new avenue for the generation of a broad spectrum of high-fidelity superhydrophobic patterns in a straightforward and low-cost fashion, requiring no vacuum processing, no hazardous organic compounds with possibilities of exploiting biodegradable or environmentally-friendly apolar polymers, rendering this technique even more technologically appealing.

## Results and discussion

**Fig. 2:**
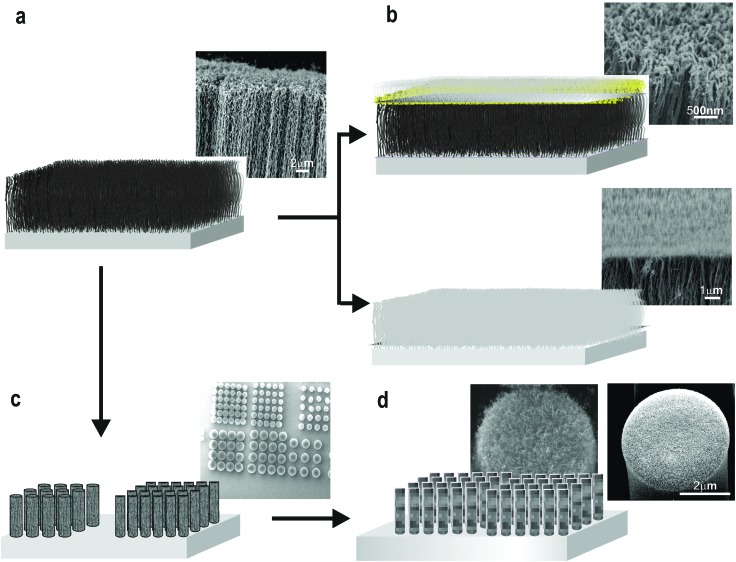
Fabrication of novel vertically aligned CNTs-based electrodes for the EHL patterning. Schematic representation and the corresponding SEM images of (a) CNTs arrays fabricated using the chemical vapour deposition process (CVD) and electron beam (e-beam) lithography combined with CVD growth process and (b) subsequently coated with a thin silicon layer to produce a range of top electrodes for EHL. Either as-grown (top) or inverted (bottom) arrays can be generated. (c) Small-diameter VACNT forests patterned into predesigned pillar structures with various dimensions and pitches are further utilized and coated with silicon (d) generating abroad range of masks for the CNT-EHL.

**Fig. 3:**
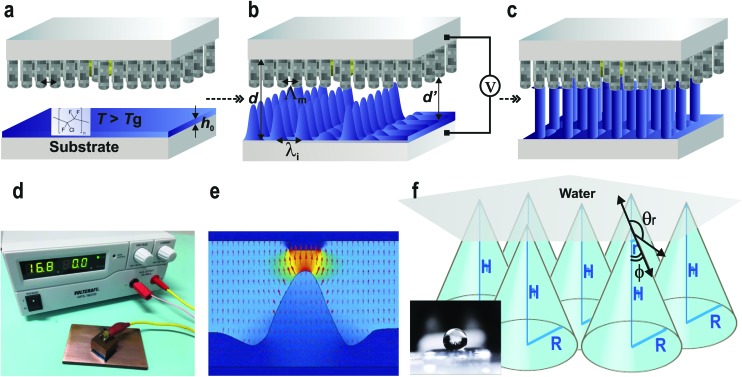
CNT-EHL based method. Placing the CNT-based top electrode above the (a) initially homogeneous thin PCTFE film with thickness, *h*_0_, (b) liquefying it above the glass transition temperature (*T*_g_) and subsequently, (c) applying a voltage, *V*, into the capacitor-like device with the controllable inter-electrode distance, *d*, triggers the amplification of a surface instability with the intrinsic film undulation wavelength, *λ*_i_. This instability, with time leads to the formation of liquid bridges between the two electrodes. The kinetics of pattern formation allows the termination of the patterning process in each stage of either the (b) cones or (c) pillars with locations predetermined by the top electrodes, at which the electrostatic pressure is the highest. The sharp tips of the VCNTs-based electrode enable to obtain the spiky replicas as well as ‘sharp’ cones. (d) A photograph of a representative experimental CNT-EHL rig consists of an assembled miniaturised capacitor device with the patterned film on the bottom electrode and the CNT-based top master electrode connected to an external voltage supply. The profile of the generated pattern depends on the ratio of *λ*_i_ and the lateral periodicity of the master top plate, *Λ*_m_. (e) Electric field distribution during EHL pattern formation.^28^ (f) Schematic of the geometrical parameters of the cone/spike like structure upon wetting and a representative optical image of a water drop on the CNT-EHL patterned surface (inset).

CNT-EHL method, elucidated below, requires assembling a miniaturised capacitor-like device comprised of a bottom electrode topped with a thin nanofilm, spun-cast from the polymer to be patterned and the topographically structured top electrode with a pattern of interest to be replicated. The details of device assembly and characterization are given in the Experimental section. The physical principles underlying the destabilization of thin films by electrohydrodynamic lithography are well-established.^25–27^ The detailed experimental configuration is schematically shown in Fig. 3. We have fabricated a range of master electrodes based on the vertical CNTs including, vertically aligned carbon nanotube (VACNT) forests both as-grown and inverted (Fig. 2a and b) as well as a range of VACNT-based micro and nano structures (Fig. 2c and d). The overall procedure for the fabrication of the CNT-based master electrodes for the EHL is illustrated in Fig. 2. Vertically oriented CNT forests were grown *via* chemical vapour deposition (CVD) process to yield well-defined stable large area substrates of nanotube arrays (Fig. 2a and b, top) or were flipped over to generate better defined, straighter CNT tips (Fig. 2b, bottom). Various VACNT site-densities in the range of 7–30% coverage grown *via* CVD process can be obtained, allowing control of the nano-gaps in the final masks (see ESI, S1†). Furthermore, VACNT-based morphologies were also designed and fabricated by prewriting the location and dimensions of CNT growth areas, generating patterns of arrays of pillars with various pre-designed height, diameters and pitches (*e.g.*, arrays with ranges of 1–5 μm diameter with a 1.5–5.5 μm pitch between the individual pillars) (Fig. 2c).

To transform the bare VACNT arrays into master electrodes, we sputter-coated the arrays with a 10 nm thin silicon layer (Fig. 2b and d). The corresponding high resolution scanning electron microscopy of the uncovered CNTs masks (Fig. 2a and c) and the low-angle backscattered SEM images of the Si coated VACNT electrodes (Fig. 2b) demonstrate uniformly covered VACNTs with a thin layer of silicon. Further, a simple silanization process enabled rendering the oxidized Si surface appolar (*i.e.*, low surface energy),^26^ and thus, led to the reduction in adhesion of the CNT-EHL patterned material to the silicon surface. Therefore, the fabricated CNT based master electrodes can be used numerous times for CNT-EHL process without undergoing any deformation or damage.

The coated VACNT arrays were then used as master top electrodes in the EHL assembly. Controlling the inter-electrode spacing (*d*), applied electric field (*E*_f_), initial film thickness (*h*_0_), patterning and termination times (*τ*_0_ and *τ*_f_) allowed fine-tuning the desired hierarchical morphologies from the polymer of choice. Since accomplishing the super-hydrophobicity of the manufactured surfaces requires a combination of both physical structure and the chemical properties, we have exploited poly(chlorotrifluoroethylene) (PCTFE) with a static contact angle, *θ*, of a water drop on a smooth film of 119 ± 3° for the EHL patterning to obtain the lowest possible surface energy properties from the final morphologies.

An overview of the CNT-EHL method is illustrated in Fig. 3. A topographically structured top electrode opposing the initially homogeneous film (Fig. 3a–d) induces high lateral electric field variations in the capacitor-like device. Since the electrostatic pressure, *p*_el_, is inversely proportional to the square of the capacitor gap, *d*, the downward protruding sharp structures of the top mask generate locations with the highest electrostatic pressure and thus, the evolving wave pattern instabilities in the thin polymer film are coupled to the heterogeneous electric field and driven upwards, eventually spanning the capacitor gap.1
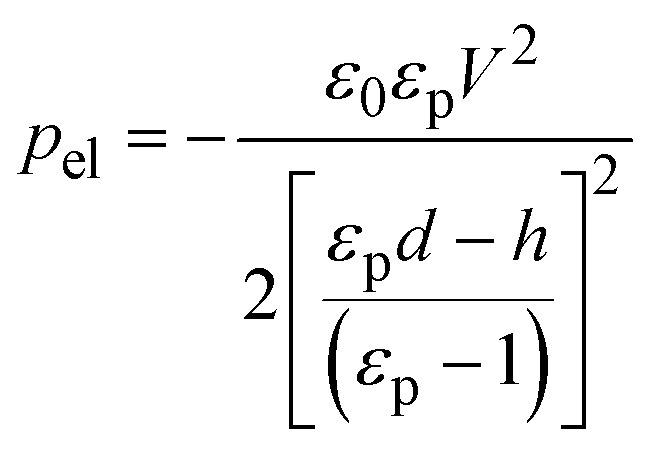



The CNT-EHL pattern replication kinetics consists of three integral parts^25,27^ including, (i) the amplification of a capillary surface instability triggered by applying an external voltage, which with time causes (ii) the formation of liquid bridges between the two electrodes (Fig. 3b and d) and eventually, (iii) this seamless sequence of capillary instability results in the coalescence of the initial capillary plugs bridging the substrates and the protruding parts of the top electrode, forming positive replicas of the imposed master pattern (Fig. 3c and d). Since the confinement of the redistributing fluid polymer morphologies is organized according to the ratio of the electrode spacing and the initial height of the polymer film in the capacitor gap, *i.e.*, the filling ratio, *f* (*f* = *h*_0_/*d*), the pattern selection during the early stage of the EHL process is a sinusoidal surface undulation and *f* determines the further stages of pattern formation. *S* is the surface area ratio of the topography, *i.e.*, fraction of the template surface that protrudes towards the polymer film. Given that the profile of the generated pattern depends on the ratio of the intrinsic film undulation wavelength, *λ*_i_ and the lateral periodicity of the master top plate, *Λ*_m_, three EHL replication scenarios are possible: (1) periodicity mismatch-small wavelength regime (*λ*_i_ ≪ *Λ*_m_), where the initial structure formation is followed by lateral coarsening of material yielding partial positive replication; (2) periodicity match-similar wavelength regime (*λ*_i_ ≈ *Λ*_m_ and *S* ≈ *f*), where positive replica of the templates is obtained and (3) periodicity mismatch-large wavelength regime (*λ*_i_ ≫ *Λ*_m_), where the pattern develops certain number of defects and every protrusion of the electrode does not faithfully generate a liquid column.

Therefore, firstly by tailoring the-designing of the top electrodes and carefully choosing the experimental parameters it is possible to control and fine-tune the patterning process. Secondly, given the ability of terminating the lithographic process at each of the pattern formation steps, it is possible to capture the individual stages of the replication kinetics of CNT-EHL and thus, the obtained structures.

To establish the precise termination times during patterning of the PCTFE into the desired morphologies, the evolving pattern formation of the sandwiched polymer was observed *via* the *in situ* imaging with an inverted optical microscope through a transparent ITO glass electrode.^25^ The electric field generated inside the micro-capacitor device causes the energetically unfavourable build-up of displacement charges at the dielectric polymer–air interface and aligns the final morphology along the field lines to lower the overall electrostatic energy. Lateral field components, which arise during intermediate stages of the EHL process lead to a cone like arrangement of the growing undulations, prior to pinning to the top electrode and reorganising into pillars (Fig. 3c). A top electrode of protruding pillars comprised of densely packed VACNT-based nanostructures generates higher electrostatic pressures at the centres of each “pillar” giving rise to spike-like, pointing cones (Fig. 3e).

**Fig. 4:**
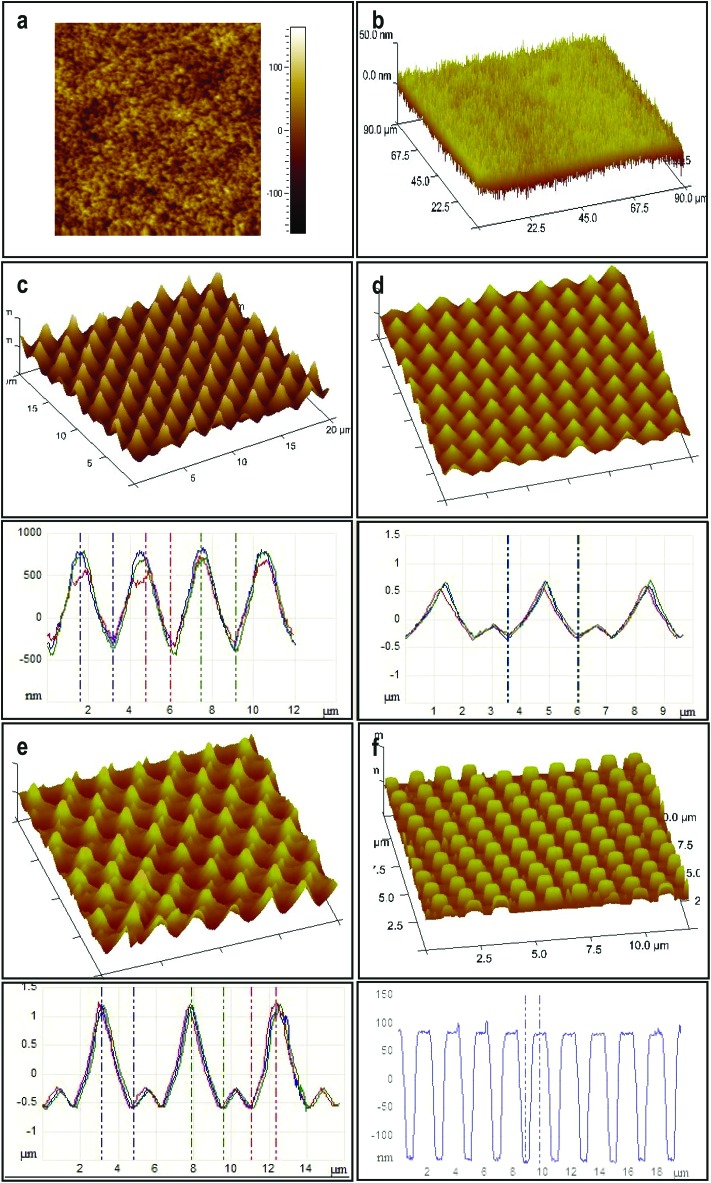
CNT-EHL replicated patterns. Atomic force microscopy height and three-dimensional images and the corresponding cross-sections of (a) curly nano-hair (CNH) surfaces, (b) of straight nano-hairs (SNH), (c) single-level spikes with rounded edges (S1L), (d) two-levelled spiky cones (S2L), (e) two-tiered heretical spiky cones (S2L2) and (f) hexagonal pillars (HP) replicated from the various imposed CNT-based electrodes.

In Fig. 4 atomic force microscopy (AFM) images and the corresponding height cross section profiles of structured surfaces obtained after the application of voltage to the capacitor device using various fabricated Si-VACNT top electrodes exhibiting a range of features are shown. When the non-patterned as-grown VACNTs-based electrodes were used, it resulted in the replication of dense nano-needles with a typical a top-surface consisting of curly nano-roughness over large substrate areas (Fig. 4a). The removal of the nonaligned nanotubes by flipping over the as-grown VACNTs (Fig. 2b, bottom) yielded better defined, straighter, nanohair-like PCTFE structures (Fig. 4b), otherwise impossible to obtain using the conventional top electrohydrodynamic electrodes.^22–24^ Imposing the VACNT-based columns with different pitches between the pillars and diameters yielded a range of morphologies, depending on the degree of master electrode periodicity match/mismatch in the presence of the laterally varying electric field, the filling ratio of the PCTFE and the corresponding patterning termination times (Fig. 4(c–f)).^27^

In the similar wavelength regime where, *λ*_i_ ≈ *Λ*_m_, a high-fidelity replica of the imposed top template was obtained terminating the process at stage (ii) of the CNT-EHL patterning, resulting in periodic cone-like PCTFE structures with a typical centre-to-centre distance of 3 μm, base diameter of 2.5 μm and the peak diameter of 0.3 μm (Fig. 4c). Using similar master electrode and initial parameters yet, terminating the CNT-EHL replication at stage (iii) of the formation kinetics, yielded well defined pillars with hexagonal packing symmetry (Fig. 4f), which have fully spanned the capacitor gap. In Fig. 4d a grid pattern of sharp 1 μm height cones with additional intermediate 300 nm height cones was replicated from an initial 93 nm thickness film (*τ* = 45 min, *d* = 230 nm, 1/*f* = 2.5, 1/*S* = 1.5). Insufficient polymer material was available to reproduce the CNT-based top electrode topography precisely. This is indicated by mismatch in the plate spacing to film-thickness ratio and the value for the lateral periodicity of the master electrode. Terminating the process just before the plugs entirely span the inter-electrode gap resulted in sharp spiky cone (top diameter of 270 nm) structures. An upper electrode patterned with features of CNTs generates a periodically modulated electrode with a charged surface giving rise to an electric field contribution which is proportional to the CNTs curvature yielding local enhancement of the electric field. Since *p*_el_ ∝ *E*_f_^2^, which is also much stronger for smaller inter-electrode distances, the instabilities which still evolve towards their final forms are focused in the direction of the highest electric field (Fig. 3e) and the peaks of the forming structures become sharper thus, terminating the pattern formation at this stage yields spiky cones.

**Fig. 5:**
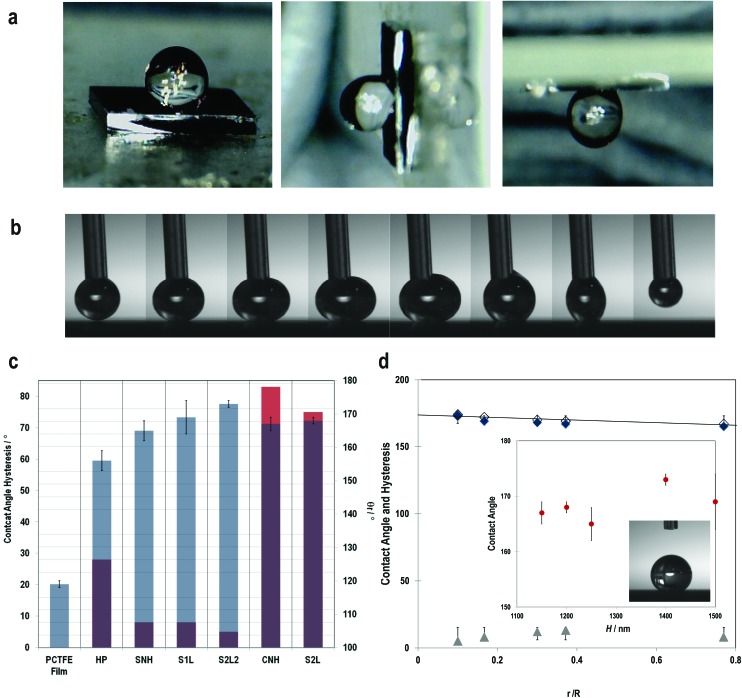
Wetting properties of the CNT-EHL fabricated surfaces. (a) Optical image sequences on the (i) flat, (ii) 90° tilted and (iii) 180° tilted CNH surface. (b) Sequence images of the water drop advancing and receding on the S2L2 surface indicating its superhydrophobic contact angle of 173°, shape of the suspending water drops and the complete receding without rupture while withdrawing the water droplet with *θ*_Adv_ ≈ *θ*_Rec_ ≈ *θ*_γ_. This behaviour corresponds to a *Cassie-Baxter* wetting state. (c) Different wetting states of all the experimental samples showing the measured contact angles (right) and the measured hysteresis (left) with SNH, S1L and S2L2 exhibiting roll-off (*lotus-leaf*) behaviour and CNH and S2L demonstrating sticky (*rose-petal*) properties not observed in the case of hexagonal pillars in comparison to the reference sample of the flat PCTFE thin film. (d) Variation of the measured contact angle (blue diamonds) and the corresponding hysteresis (grey triangles) with the geometric parameters is in agreement within their error margins and are well described by the theoretical prediction (line). The theoretical data follow the same trend, but are offset towards slightly higher values. Inset: Variation of the contact angle as a function of the structures’ height. Over a height variation of 350 nm, the contact angle changed by only 22% compared to a variation as function of *r*/*R* by a factor of 7.

Using the as-grown CNTs-based master electrode yielded curly nano-hair (CNH) like roughness, with shallow but sharp peaks and valleys from the patterned PCTFE film, generating a superhydrophobic surface which exhibited the *rose-petal* effect with a contact angle of 167 ± 2° and strong adhesive forces, with a typical contact angle hysteresis of 83 ± 2° (Fig. 4a and 5a). On the other hand, the inverted CNT forest based top electrodes yielded well-defined, dense straight nano-hair (SNH) like morphology establishing hierarchical nanoscale structure (intra and inter CNTs) mimicking the *lotus-leaf* behaviour (Fig. 4b and 5b) with a contact angle of 165 ± 3° and a hysteresis of 8 ± 2°.

SNH fluorine terminated hydrophobic polymer exhibits considerably increased water repellent properties due to the large surface contact area between liquid droplet and the nanohair-like surface. Interestingly though, on the mesoscopic scale the PCTFE patterned film appears mostly smooth. In a densely packed porous network of CNH-roughness with minute variations in heights, upon wetting with a water drop a thin layer of liquid is left behind and the solid fraction in contact with liquid is increased with the network being penetrated by water, yielding the high solid–water adhesion and therefore, high contact angle hysteresis. On the other hand, closely packed needle like SNH nanostructure restrains the droplet spread, leading to a smaller contact angle hysteresis and roll-off super-hydrophobicity. However, the surface roughness must still be maintained, too dense a structure will slowly close the porous network, thus decreasing the water repellence. Since the surface roughness of the VACNT forests can be controlled, (see ESI, S1†) it is possible to also tune the degree of the apolar properties for the CNT-EHL replicated structures. While the curly-like nano-roughness with shallow peaks and valleys exhibits *rose-petal* like behaviour creating a thin water layer upon wetting, the sharp needle-like surface with measured contact angle of 165° and the contact angle hysteresis of 8°, inhibits liquid from remaining on the surface behaving like a *lotus-leaf* surface.

For the structures shown in Fig. 4c, generated *via* CNT-EHL, we measured higher contact angles of 169° ± 5° than for the forest like nanorough surfaces and contact angle hysteresis of 8° ± 2° resulting in low adhesive forces between the liquid and the surface, allowing the water drop to roll-off the substrate easily. This surface is comprised of hexagonally packed cones with nanoscale, slightly rounded triangular peaks which exhibit contact angle hysteresis <10°, resulting in a non-wetting of the microstructures’ spaces between the spikes. The air trapped in-between the spikes yields a heterogeneous surface comprised of both air and solid. The hierarchical dual structure of tapered cones in Fig. 4d yielded highly apolar surfaces (CA = 168 ± 1°) along with the sticky properties (hysteresis of 75 ± 2°), mimicking the *rose-petal* effect.

The physical principles underlying the wetting theory are well understood.^4–7^ Here, we briefly summarize the principles of the wetting and superhydrophobicity on flat and rough surfaces (see ‘ESI’, S2† for the more detailed background on the wetting theory). For flat surfaces, the Young's Equation is given by:

2 *γ*_SV_ = *γ*_SL_ + *γ*_LV_ cos *θ*

where, *γ* is a surface energy and *θ* is a contact angle (CA). For wetting on rough surfaces where the contact angle is larger than 90°, it is energetically more favourable for the liquid drop to wet a smaller area to reduce the total interface energy, *γ*_SV_ < *γ*_SL._ In particular, above a critical roughness value, a cone structure with the contact area for a known vertical force is given by: *A* ∝ [sin *φ* cos^2^(*θ* – *φ*)]^–1^ where, *φ* is the fraction of the projected area of the solid surface that is wetted by liquid, it is energetically more favourable for the water drop to contact only a very small fraction of the surface asperities and move up the cone structure. Therefore, sharp coned CNT-EHL generated structures are favourable geometries to obtain superhydrophobic surfaces since their sharp tips enable the minimum solid–liquid contact with maximum vertical force per contact area as demonstrated in Fig. 4e, where a structured surface yields a contact angle of 173 ± 1° and a hysteresis of 5 ± 2°. These physical structures in combination with chemical properties of the cone-structured polymers lay the platform for optimal surface topography, which in turn gives rise to the super-hydrophobicity. Furthermore, these sharp and rounded cone surfaces closely resemble in their morphologies and dimensions found in the structures of natural systems, as can be clearly seen from the SEM images of Fig. 1a–c.

The contact angle of rough surfaces (*i.e.*, cone structures and CNT-like surfaces) with *rose-petal* and *lotus-leaf* effects can be extracted from the modified *Cassie-Baxter* equation:

3a cos *θ*_*r*_ = *ρf*_s_cos *θ* – *f*_v_

where, *θ*_*r*_ is the apparent contact angle of the micro- and nano structured surfaces, *f*_s_ is the fraction of the areas occupied by the solid–water interface and *f*_v_ is the fraction that correspond to the vapour gaps, *θ* is the Young's contact angle and *ρ* is the roughness factor, calculated from triadic curve for fractal geometry^29^

3b *ρ* = (*R*/*r*)^*D*–2^

For a three-dimensional space *D* ≈ 2.2618 and *R* and *r* are the upper and the lower limits of the surface topographies.^29,30^ Based on the surface geometry considerations described in Fig. 3f, the Casie–Baxter equation can be reformulated (see ESI, S3†) exhibiting the evident dependence on the hexagonally packed cone geometry:4
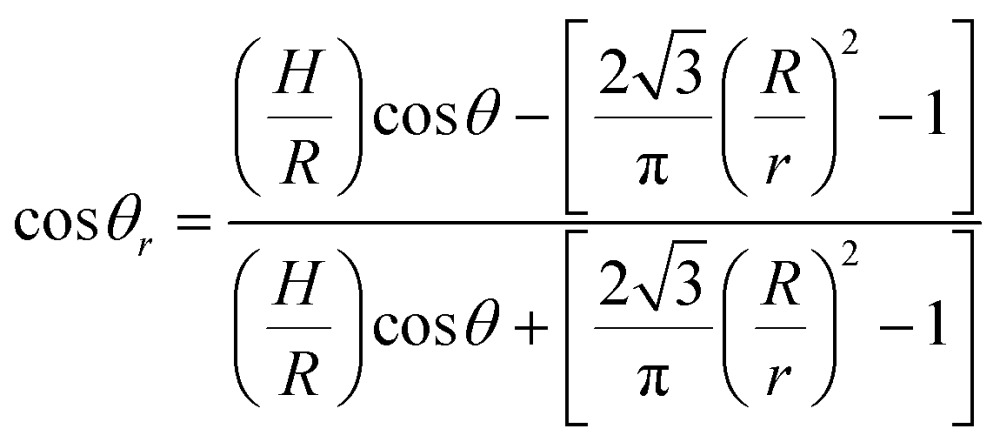



For a hexagonal array of cones (Fig. 4c–e), the area fraction of the solid surface that is in contact with the liquid is given by^4–7^
5
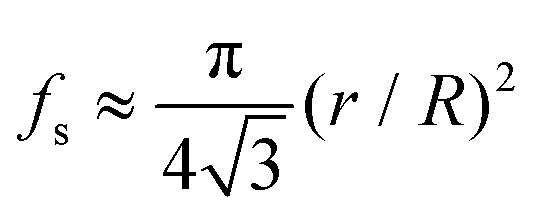



For the surface morphologies shown in Fig. 4a and b, and only considering the top of the nano-hair like surfaces roughness, the theoretical contact angle can be extracted from the eqn (3a), (3b) and (5). Fig. 5 shows the experimental contact angles and hysteresis on the fabricated surfaces as well as the function of morphological geometric parameters. The effects of structure morphologies and dimensions, fabricated *via* the CNT-EHL, on the water contact angle and hysteresis, both experimentally measured and theoretically calculated, are summarised in Table 1.

**Table 1:** Geometrical effects of the CNT-EHL generated surfaces on the contact angle and hysteresis (Hys). The experimental results in comparison to the theoretical predictions as function of varying surface roughness

Surface	*R*/nm	*r*/nm	*H*/nm	*θ*_*r*_/°	STDV *θ*_*r*_	Hys/°	STDV Hys	Property	Calculated *θ*_*r*_/°
PCTFE film	—	—	100	119	1	—	—	—	—
HP	1750	—	250	155	3	28	7	Wenzel	156
SNH	1.3	1	1250	165	3	8	1	*Lotus*	167
S1L	2500	300	1500	169	5	8	2	*Lotus*	174
S2L2	3000	300	1400	173	1	5	3	*Lotus*	174
CNH	3.5	1.3	1150	167	2	83	2	*Petal*	169
S2L	1000	300	1200	168	1	75	2	*Petal*	170

It can be clearly seen that the wetting behaviour is considerably enhanced by the surface structure and dimensions in comparison to the flat surfaces of the same material. Super apolar behaviour is demonstrated from all the CNT-EHL patterned structures with contact angles exceeding 160° except the cylindrical pillars geometry (Fig. 5c) and the S2L2 exhibiting the most superhydrophobic properties with a contact angle as high as 173°. Nevertheless, while SNH, S1L and S2L2 structures exhibit roll-off superhydrophobicity mimicking the *lotus-leaf* like behaviour (Fig. 5b and c) in the case of CNH and S2L structures, water appears to penetrate a few nanometres into the voids and therefore, facilitates strong adhesive forces combined with the superhydrophobicity, mimicking the *rose-petal* effect (Fig. 5a and c).

Whereas for the hierarchical S2L structures both levels of roughness contribute to the pinning effect, with complete wetting on the larger spikes and the smaller cones which contribute to the second order of roughness with the air trapped within, in the case of the S2L2 surface, where similar morphology is observed, the second order roughness of the small spikes is considerably lower than the primary cones and therefore, their effect is negligible.^31^ According to Cassie-impregnating wetting regime, the liquid wets the larger-scale structure and penetrates the smaller nanoscale valleys and therefore the adhesive force between the water drop and the surface is very high supporting the droplet even when the surface is tilted at an angle or turned upside down (Fig. 5a). In addition defects and artifacts on the mesoscale (micro-to nano) have been shown to yield either the high localised surface energy, possibly penetrating into the static drop increasing the adhesion forces resulting in sticky surfaces on the macroscale.^14,32^ The degree of opening angle of the cone structures has been also shown to influence the transition from the slippery to sticky behaviour.^14,33^

It is evident from Fig. 5d that the contact angles as a function of the geometric parameters of the surface roughness of the CNT-EHL patterned surfaces have a linear dependence, indicating that the larger the fractional contact area, the smaller the apparent contact angle (Fig. 5d). As qualitatively extracted from Fig. 5a and b the marked structural height has only a minor influence on the contact angle. This is quantitatively shown in the inset of Fig. 5d, where *θ*_*r*_ changed by only 22% over height variation, despite a relative variation of the water penetration by a factor of 7 indicating a considerably smaller influence of the height on the superhydrophobicity in comparison to the surface roughness factor.

Finally, our results highlight the importance of the ratio of the surface roughness factor and heterogeneity parameters, *R*_s_ for tuning the superhydrophobic properties. Typically, the ratio of the roughness factor as well as the fractional area of contact can be calculated by averaging the surface roughness over a given area which is smaller than the liquid droplet. The contact angle hysteresis is influenced by the value of the surface roughness which itself affects the ability of the interface to pin the triple line and therefore, decreasing the *R*_s_ relative to the liquid drop size eventually rendering the contact angle hysteresis of a negligible value. For the hexagonally packed cone structures, the dependence of the surface roughness in Fig. 5(c and d) implies that the triple-phase contact length has a linear dependence on the contact angle hysteresis (see ESI, S4†).

In summary, we have demonstrated an innovative, controllable and facile yet scalable method to fabricate a broad range of super apolar surface morphologies with a single or multi-level hierarchy. Vertically aligned carbon nanotube forests were exploited in an unconventional way to produce a range of robust lithographic electrodes, further exploited in conjunction with electrohydrodynamic patterning. A range of configurations was produced by verifying the top electrode design and the experimental parameters during the CNT-EHL patterning process yielding various hierarchical architectures, mimicking the *lotus-leaf* and *rose-petal* like surface morphologies and properties. The generated hierarchical structures enable enhancing the hydrophobicity *via* different length scales of roughness. When the water can penetrate the larger-scale texture, but cannot enter into the smaller structures, the patterned surface effectively mimics the ‘*lotus-leaf*’ effect. However, when the larger micro- and nanostructures are impregnated by water this gives rise to high solid–water adhesion and therefore high contact angle hysteresis. The tuneable wetting properties can be easily switched between the various behaviours including the sticky or roll-off superhydrophobicity and super-hydrophilic surfaces can also be fabricated upon demand from a simple switch to alternative polymers and morphologies. Furthermore, thin EHL patterned films can be easily floated off the supporting substrates and transferred on different support surfaces and therefore, can be used as advanced superhydrophobic coatings which conformably adhere to underlying substrates with any dimension and morphology. The versatility of the CNT-EHL technique renders it easily extendable for a broad range of more intricate, adjustable geometrical microstructures enabling direct biomimetics of nature's unique wettability. Vertically aligned carbon nanotube forests patterned into predesigned structures can be also utilized for additional applications including for instance, straightforward and cost-effective substrates for high-throughput multiplex detection. Moreover, because vertically oriented CNTs exhibit functionalities such as electrical conductivity and unique adsorption properties, these can be further harnessed in their development as novel chemical and bio-sensing platforms. In this tuneable mode, CNT-EHL is a promising prospect for the robust, straightforward, and low-cost fabrication of sub-micrometer patterned substrates to facilitate a plethora of low energy surfaces for coatings, fabrics and microfluidic device technologies with high mechanical durability and optical transparency.

## Experimental

### Growth of the vertically aligned carbon nanotube forests

CNT forests were grown on the 5 × 5 mm^2^ silicon wafers, using the cold-wall system of the catalytic chemical vapour deposition (CVD) process.^34^ Substrates were sputter-coated with a catalyst layer consisting of Al_2_O_3_ buffer and iron catalysts. During the growth process, initially 500 sccm of H_2_ was heated to 750 °C at 5 min under controlled system pressure of 15 mbar. CNTs growth proceeded at 750 °C with a gas flow of H_2_ : C_2_H_2_ (460 : 40 sccm). Upon the completion of the growth, the substrate was cooled to room temperature under the flow of 500 sccm of hydrogen.

### Fabrication of patterned CNT arrays

CNTs arrays were generated using an electron beam lithography combined with CVD growth process, where initially, a layer of photoresist was spin-coated on a silicon wafer which was further annealed at 120 °C for 2 minutes. Consequently, the resist was exposed under the electron beam with (pre)-written dimensions. Finally, it was post-baked at 140 °C for 2 minutes and developed in CD26 for 30 s, and the CNT-based pillar arrays were obtained. These structures were further filled by depositing 10 nm alumina and 1.3 nm of iron through a sputtering process, followed by lifting-off the resist with acetone. CVD process was utilised using a combination of H_2_ : C_2_H_2_ (70 : 30 sccm) at 750 °C for 2 minutes. CNTs arrays with desired dimensions and pitches were eventually grown on top of the patterned catalysts.

### Fabrication of the inverted CNT forests

Initially, a homogeneous poly(methyl methacrylate) film was spin-cast on a silicon substrate, followed by placing the VACNTs facing the PMMA layer and annealing at 180 °C for 1 minute. The substrate was then cooled down *t* below the glass-transition temperature of the polymer film, resulting in a solidification of the film while embedding the upper ends of the CVD-grown CNTs forest. The VACNTs were subsequently peeled off the original silicon wafer, exposing well-defined straight tips.

### CNT-EHL patterning

The fabricated CNT-based electrodes were coated with a silicon layer (Kurt J. Lesker Si sputtering target 99.999% purity). To ensure the reusability of the CNT-based electrodes, these were rendered hydrophobic by the deposition of a 1,1,1,2*H*-perfluorodecyltrichlorosilane self-assembled monolayer to reduce the adhesion between the mask and the EHL patterned polymer. Alternatively, a non-stick self-assembled monolayer was deposited from liquid octadecyltrichlorosilane phase. Silanization was performed by immersion of the substrate in the freshly prepared silane solution (0.25% OTS in hexadecane).

Highly polished p-doped silicon (Si) wafers, with <100> crystal orientation (Wafernet Gmbh, Eching, Germany) covered by 100 nm thick silicon oxide layer were used as substrates. Initially, the substrates were cleaned in a ‘Piranha’ solution consisting of 3 : 1 H_2_SO_4_ (98%) : H_2_O_2_ (30%), followed by thorough rinsing with deionised water and dried under N_2_. Thereafter, silicon wafers were cleaned using a snow-jet gun immediately before film deposition and capacitor patterning assembly. Transparent indium–tin oxide (ITO) covered glass slides with a resistivity of 80 cm^–2^ were also used as substrates, allowing the *in situ* optical tracking of the pattern formation or replication process. Thin films of PCTFE (Young's modulus 2.7 × 10^9^ Pa, *ε* = 2.6, *T*_g_ = 103 °C, density = 1 g mL^–1^ at 25 °C, intrinsic viscosity, [η] = 0.70) were spin-coated onto a silicon wafer with typical concentrations of 2–3% polymer by weight. Facing it, a top electrode comprised of the silicon coated VACNT (as-grown and inverted) forests and arrays, was mounted at a specific distance, leaving a thin air gap, *d*. The silicon wafers were electrically contacted by evaporating a 10 nm chromium layer, followed by a 100 nm gold layer on the unpolished backside. When ITO glass was used as bottom electrodes, these were contacted by scratching the polymer film at two corners before applying the silver paste.

The experiment was initiated by liquefying the spin-cast PCTFE films by annealing above the softening/glass transition temperature (typically to around 130 °C) of the polymer while the voltage (between 40–70 V) was applied to the electrodes and subsequently, cooling sample to RT solidified the polymer before the voltage was removed, terminating the patterning process (typically 20 h). A laterally varying electric field density was introduced to the system by mounting a topographically structured CNT-based master electrode onto the polymer film. Expressed by the ratio between the intrinsic wavelength *λ*_i_ and the lateral periodicity (or lateral size of nonperiodic structures) *Λ*_m_ of the master structure, three replication cases are described: (i) *λ*_i_ < *Λ*_m_ (ii) *λ*_i_ ≈ *Λ*_m_ and (iii) *λ*_i_ > *Λ*_m_. After freezing-in the samples by reducing the temperature to room temperature, the electric field was disconnected and the upper electrode was removed. Pattern replication was monitored and recorded by a microscope and a connected computer throughout the experiment. After removal of the top electrode, the quenched polymer film was further characterized by the atomic force microscopy.

### Characterisation

AFM measurements were performed using a Nanoscope IV Dimension 3100 (Veeco Instruments Inc.) microscope operated in tapping mode using the NSG 20 cantilevers with a resonance frequency of 260 kHz and a stiffness of 28 N m^–1^. Image processing and analysis was carried out with the instrument's software version V612r2 and V530r2. AFM measurements yielded the geometric dimensions of the CNT-EHL structure including, the aspect ratio, the pitch between the generated morphologies, their heights and diameters. Contact angle and hysteresis were measured at 3–5 different areas on each sample using a computer controlled telescopic goniometer (KSV CAM 200) with digital image acquisition. A numerical fitting algorithm was applied to determine the advancing and receding contact angles from the side-view of drops. Static contact angles are measured on drops with a volume between 5 μl < *V* < 20 μl. The drop pictures were fitted using the contact angle goniometer software. For static contact angles, the Young–Laplace fitting algorithm was used and a base-line tilt was allowed. Dynamic contact angles were measured by continuously increasing or decreasing the size of a drop on a surface. The increasing and decreasing speed was between 0.5 μl s^–1^ < *S*_drop_ < 2 μl s^–1^. The images of the advancing and receding drops were analysed using ImageJ (Drop_Analysis Package). The scanning electron microscopy (SEM) measurements were performed using a LEO ULTRA 55 SEM including a Schottky emitter (ZrO/W cathode) at acceleration voltages of 1–5 kV with a lateral resolution of 2–5 nm. Low-angle backscattered electron imaging mode was used to contrast the noncoated VACNTs and those sputtered with silicon, providing the atomic number contrast.
