# Supplementary material for: Tunable superapolar Lotus-to-Rose hierarchical nanosurfaces via vertical carbon nanotubes driven electrohydrodynamic lithography
Source: Nanoscale. 2017 Jan 4;9(4):1625–36. doi: 10.1039/c6nr08706j (PMC5433428; doi:10.1039/c6nr08706j)
Supplement: Supplementary file 1 [file NR-009-C6NR08706J-s001.pdf]

Electronic Supplementary Material (ESI) for Nanoscale.
This journal is © The Royal Society of Chemistry 2017

Tunable Superapolar *Lotus-to-Rose* Hierarchical Nanosurfaces *via* Vertical Carbon Nanotubes Driven Electrohydrodynamic Lithography

-Supporting Information-

Chiara Busa[1], Jonathan James Stanley Rickard [1, 2], Eugene Chun [1], Yaw Chong[1], Pola Goldberg Oppenheimer [1,]*

[1] School of Chemical Engineering, University of Birmingham, Birmingham B15 2TT, UK
[2] Department of Physics, Cavendish Laboratory, University of Cambridge, Cambridge CB3 0HE, UK

*Corresponding E-mail: GoldberP@bham.ac.uk

S1: Variable VACNT Forests' Density

Carefully monitored CVD process allows generating a range of site densities of the VACNTs between 7% and 30% and thus, enables to control of the nano-gaps in the final masks used for the CNT-EHL. To evaluate the site-densities, liquid-induced compaction method was used. Isopropanol was added to the samples and been allowed to dry leading to collapse of the CNT forests as shown in the Figure S1. The percentage of the aggregated CNTs was the calculated by the fraction of number of pixels of the aggregated forest areas divided by that of the entire image.

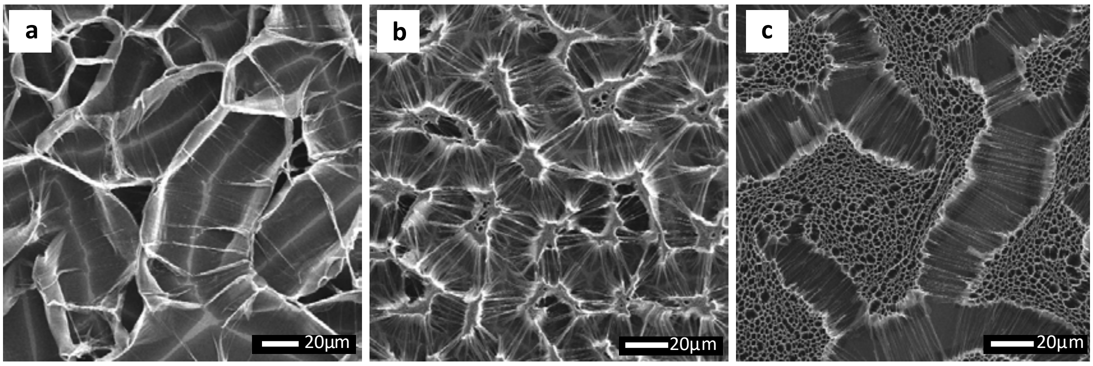

Figure S1: Top view SEM images of coagulated CNTs forests following the liquid-induced densification by isopropanol. (a-c) CNTs with various densities (10^{10}-10^{12} cm^{-2}) and coverage of 7%, 15% and 30%, respectively.

S2: Young's Equation and Classical Wetting Theory

On an ideal flat surface the contact angle is described by the equation, formulated by Young, which demonstrates that contact angle is a property directly dependent on the surface tension between the three interfaces (Fig. S2):

$$cos\theta = \frac{\gamma_{SV} - \gamma_{SL}}{\gamma_{LV}}$$

where,
θ – Water contact angle
γ – Surface tension
SV, LV, SL – Solid-Vapour, Liquid-Vapour and Solid-Liquid interfaces, respectively.

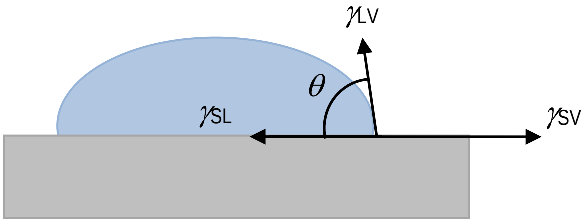

Figure S2. Schematic representation of forces at the three phase contact line for a droplet.

According to this equation a hydrophobic material has an inherent water contact angle above 90° and a hydrophilic material has an inherent water contact angle below 90°. The wetting behaviour on rough surfaces however, is more complex than described by the Young's theory. It has been established that the surface roughness enhances the inherent wetting properties of the materials. [*Ind. Eng. Chem.,* **1936**, 28, 988; *Trans. Faraday Soc*.**1944**, 40, 546] While Wenzel's theory is established on complete contact between the liquid and the surface and for hydrophobic materials there is no gap between the droplet and the surface with the contact angle θ > 90°, in the Casie-Baxter's model the small contact area between the liquid and the solid phase and the resulting small contact angle hysteresis allow drops to easily 'roll' off a surface due to the presence of air-pockets under the droplet.

S2.1: Wenzel's Model

Wenzel's wetting is strongly dependent on the surface roughness, which is a measure of the true against the projected surface area:

$$\cos\theta^{W} = r\cos\theta$$

where,

θ^{W}- Apparent contact angle

θ – Young/Equilibrium contact angle

r = Roughness ratio

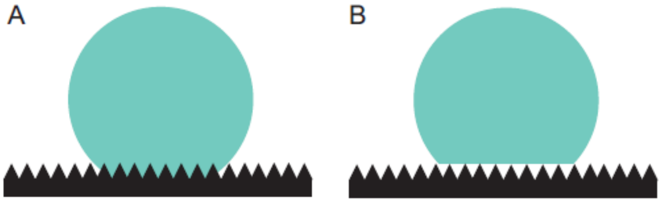

Figure S3. Wetting on hydrophobic rough surfaces with a (A) *Wenzel* drop and with a (B) *Cassie-Baxter* drop.

S2.2: Cassie-Baxter Model

In the case when the contact angle is above 90° and there is a thin air-filled gap between the drop and the surface with high surfaces roughness, it is energetically more favourable for the hydrophobic Cassie-Baxter regime to prevail.

Cassie-Baxter wetting is characterised by the drop located on the peaks of the surface structures which does not penetrate into the deeper holes. This air-pocket state corresponds to partial non-wetting of the surface, which is the most favourable solution for the energy equation for very rough hydrophobic materials:

$$cos\theta^{C} = \varphi_s(cos\theta) + (1\text{-}\varphi_s)\ cos\theta_X$$

where,

φ_s - Fraction of surface available at the top of protrusions

θ_X – Contact angle with the vapour in the gaps (usually taken as 180°)

Cassie-Baxter model is often called the superhydrophobic wetting and it has been observed by Barthlott *et al.* on the surface of *Nelumbo Nucifera*, commonly known as the 'Lotus Leaf effect'. Superhydrophobicity has been studied on various surfaces, including for instance, periodic structured micropillars, biomimetic superhydrophobic structures, carbon-nanotubes based surfaces, alumina nanowires, silicon 'nanograss' and fibrous textile surface structures. [*Soft Matter*, 2005, 1,55; *Microelectronic Engineering*, 2005, 78; *Langmuir*, 2006, 22,5998; *Langmuir*, 2008, 24,1959; *Soft Matter*, 2008, 4, 224; *Chemical Communications*, 2009, 1043; *Advanced Materials*, 2007, 20.]

S2.3: Cassie Impregnating Wetting State

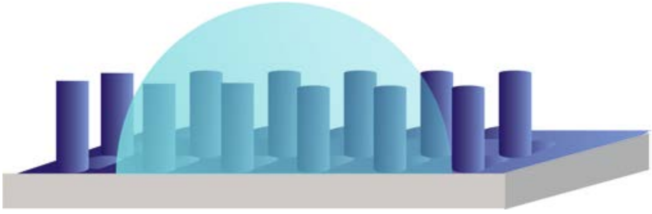

Figure S4. Schematic representation of Cassie-Impregnating wetting state.

The impregnating state, depicted in Figure S4 can be determined using the following equation:

$$cos\theta^{C} = 1 - f_s + f_s cos\theta$$

where, f_s is the solid fraction underneath the liquid drop. However, for this Cassie impregnating state to be viable, it must satisfy:

$$cos\theta = \frac{1 - f_s}{r - f_s}$$

A classic example in nature of a Cassie impregnating regime is the rose petal, also known as the 'Rose Petal Effect'`. The water film is able to impregnate the surface by seeping into the larger crevices, but not the smaller ones. Therefore, the water drop depending on its size can attach to the surface due to the very high adhesive forces.

S2.4: Combined Model

An additional possibility exists when a liquid droplet may not bridge the gap on a rough surface completely. Unlike in Wenzel's model, where liquid film must be present before droplet is placed on the surface, there is an initially dry rough surface. Without a liquid film, the drop might spread below the flat surface, as occurs on surfaces such as sol-gels and soils, where internal spreading can occur rapidly, resulting in a combined Wenzel's and the Cassie-Baxter model., which gives the effective macroscopic water contact angle for Cassie-Baxter wetting [*Trans. Faraday Soc*.1944, 40, 546]

$cos\ \theta_{CB} = f\ cos\ \theta_{Y} + f - 1.$

The roughness of the fraction of the surface in contact with the liquid, r_f also controls the surface wetting properties, and a more specific definition of the air-pocket state can be given by:

$cos\ \theta_{CB} = r_f f cos\ \theta_Y + f - 1$.

Ishino *et*. al have theoretically studied the cross-over between *Wenzel* and *Cassie-Baxter* wetting [*Europhysics Letters*, 2004, 68, 419] for pillar arrays and suggested that the penetration versus air-pocket state wetting depends strongly on the roughness r_f and the solid fraction f of the surface structures. The roughness of liquid−solid contact was further calculated according to the threshold value of slope and the hemispheric top model taking into the consideration the roughness of both micro and nano structures. [*Langmuir,* 2015, 31, 10807] The contact angle of rough surfaces (*i.e.,* cone structures and CNT-like surfaces) with *rose-petal* and *lotus-leaf* effects can be extracted from the modified *Cassie-Baxter* equation, as described in on Pg10 of the manuscript:

$$cos\theta_r = \rho f_s cos\theta - f_v$$

where, θ_r is the apparent contact angle of the micro- and nano structured surfaces, f_s is the fraction of the areas occupied by the solid-water interface and f_v is the fraction that correspond to the vapour gaps, θ is the Young's contact angle and ρ is the roughness factor, which can be simplistically calculated from triadic curve for fractal geometry [*Advanced Materials*, 2002, 14, 1857]:

$$\rho = (R/r)^{D-2}$$

For a hexagonal array of cones, the area fraction of the solid surface that is in contact with the liquid is given by $f_s \approx \frac{\pi}{4\sqrt{3}}(r/R)^2$.

For instance, for S2L structure with r = 300nm, R =1000nm (see Fig. 4), f_s = 0.040, f_v= 0.96, based on the modified *Cassie-Baxter* equation:

$cos\theta_r$ = 0.040 x cos(119°) - 0.96= -0.9792 and therefore, θ_r = 168.2°.

For the surfaces topographies shown in Figure 4a, the solid fraction of CNH structures is 0.055 (only the top of the nano-hair like surfaces roughness is considered), R = 3.5nm. r = 1.3nm,

Therefore, $\rho = (R/r)^{D-2} = (3.5/1.3)^{0.2618} = 1.29$

$cos\theta_r$ = 1.29 x 0.055 x (-0.48) and thus, θ_r= 167°

S3: *Casie-Baxter* Equation in terms of Structures' Geometrical Parameters

For the hexagonal packing geometries, typically generated during the EHL process, the Casie-Baxter equation:

$cos\theta_r = f_s cos\theta - f_v$

can be reformulated taking into account the geometrical parameters:

$$f_v = (2\sqrt{3}H^2 - \pi h^2)\frac{r^2}{h^2 + r^2}$$

$$f_s = \frac{\frac{\pi h^3 r}{h^2 + r^2}}{\frac{\pi h^3 r}{h^2 + r^2} + \frac{2\sqrt{3}H^2 r^2}{h^2 + r^2} - \frac{\pi h^2 r}{h^2 + r^2}} = \frac{\pi h^3 r}{\pi h^3 r + 2\sqrt{3}H^2 r^2 - \pi h^2 r} = \frac{\pi h^3}{\pi h^2(h - r) + 2\sqrt{3}H^2 r}$$

$$= \frac{h}{(h - r) + \frac{2\sqrt{3}}{\pi}(\frac{H}{h})^2 r}$$

$$cos\theta_r = \frac{h}{(h - r) + \frac{2\sqrt{3}}{\pi}\left(\frac{H}{h}\right)^2 r} cos\theta - \left[1 - \frac{h}{(h - r) + \frac{2\sqrt{3}}{\pi}\left(\frac{H}{h}\right)^2 r}\right] =$$

$$= \frac{h}{(h - r) + \frac{2\sqrt{3}}{\pi}(\frac{H}{h})^2 r} cos\theta - \frac{\frac{2\sqrt{3}}{\pi}\left(\frac{H}{h}\right)^2 - 1}{\frac{h}{r} + \frac{2\sqrt{3}}{\pi}\left(\frac{H}{h}\right)^2 - 1}$$

$$= \frac{\frac{h}{r}}{\frac{h}{r} + \frac{2\sqrt{3}}{\pi}(\frac{H}{h})^2 r - 1} cos\theta - \frac{\frac{2\sqrt{3}}{\pi}\left(\frac{H}{h}\right)^2 - 1}{\frac{h}{r} + \frac{2\sqrt{3}}{\pi}\left(\frac{H}{h}\right)^2 - 1}$$

$$cos\theta_r = \frac{\left(\frac{h}{r}\right)cos\theta - [\frac{2\sqrt{3}}{\pi}\left(\frac{H}{h}\right)^2 - 1]}{(\frac{h}{r}) + [\frac{2\sqrt{3}}{\pi}(\frac{H}{h})^2 - 1]}.$$

Resulting in:

$$cos\theta_r = \frac{\left(\frac{H}{R}\right) cos\theta - [\frac{2\sqrt{3}}{\pi}\left(\frac{R}{r}\right)^2 - 1]}{(\frac{H}{R}) + [\frac{2\sqrt{3}}{\pi}(\frac{R}{r})^2 - 1]}$$

S4: Triple Line Length *vs* the Contact Angle Hysteresis

For rough superhydrophobic surfaces, Schneider *et* al. [*Scientific Reports*, 2016, 6, 21400] have recently demonstrated the importance of using the triple line length, ω_{tf} over the drop's diameter. For cone structures with hexagonally symmetry:

$$\omega_{tf} = 2r\left(\frac{1-\frac{Rh}{H}}{\frac{RH}{H}} + 1\right) = 2r\left(\frac{1}{R} - \frac{h}{H} + 1\right) = 2r(\frac{1-r}{R} + 1)$$

Plotting the dependence of the cones' surface roughness in Figure S5 reveals that the triple-phase contact length has a linear dependence on the contact angle hysteresis.

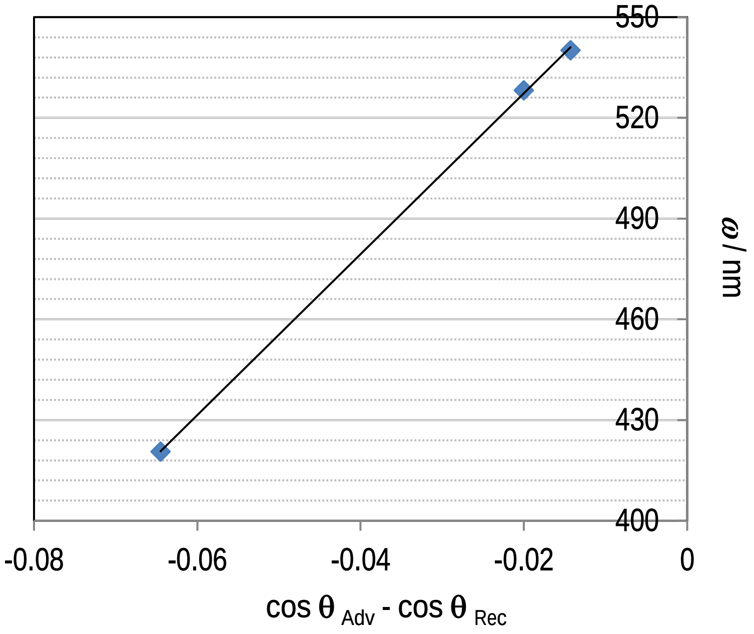

Figure S5: Triple-line length *versus* the contact angle hysteresis dependence for cone structures.
